# Pyrogenic and Precipitated Amorphous Silica Nanoparticles Differentially Affect Cell Responses to LPS in Human Macrophages

**DOI:** 10.3390/nano10071395

**Published:** 2020-07-18

**Authors:** Massimiliano G. Bianchi, Martina Chiu, Giuseppe Taurino, Roberta Ruotolo, Nelson Marmiroli, Enrico Bergamaschi, Francesco Cubadda, Ovidio Bussolati

**Affiliations:** 1 Department of Medicine and Surgery, University of Parma, 43126 Parma, Italy; martina.chiu@unipr.it (M.C.); giuseppe.taurino@unipr.it (G.T.); 2 Department of Chemistry, Life Sciences and Environmental Sustainability, University of Parma, 43124 Parma, Italy; roberta.ruotolo@unipr.it (R.R.); nelson.marmiroli@unipr.it (N.M.); 3 The Italian National Interuniversity Consortium for Environmental Sciences (CINSA), University of Parma, 43124 Parma, Italy; 4 Department of Public Health Sciences and Pediatrics, University of Turin, 10126 Turin, Italy; enrico.bergamaschi@unito.it; 5 Istituto Superiore di Sanità-Italian National Institute of Health, 00161 Rome, Italy; francesco.cubadda@iss.it; 6 Microbiome Research Hub, University of Parma, 43124 Parma, Italy

**Keywords:** amorphous silica nanoparticles, autophagy, E551, endotoxin, food additive, glutamine synthetase, immunometabolism, inflammation, macrophages

## Abstract

Previous work has demonstrated that precipitated (NM-200) and pyrogenic (NM-203) Amorphous Silica Nanoparticles (ASNPs) elicit the inflammatory activation of murine macrophages, with more pronounced effects observed with NM-203. Here, we compare the effects of low doses of NM-200 and NM-203 on human macrophage-like THP-1 cells, assessing how the pre-exposure to these nanomaterials affects the cell response to lipopolysaccharide (LPS). Cell viability was affected by NM-203, but not by NM-200, and only in the presence of LPS. While NM-203 stimulated mTORC1, neither ASNPs activated NFκB or the transcription of its target genes *PTGS2* and *IL1B.* NM-200 and NM-203 caused a block of the autophagic flux and inhibited the LPS-dependent increase of Glutamine Synthetase (GS) expression. Both ASNPs suppressed the activation of caspase-1, delaying the LPS-dependent secretion of IL-1β. Thus, ASNPs modulate several important pathways in human macrophages, altering their response to LPS. NM-203 had larger effects on autophagy, mTORC1 activity and GS expression than NM-200, confirming the higher biological activity of pyrogenic ASNPs when compared with precipitated ASNPs.

## 1. Introduction

As the additive E551, Food-grade Amorphous Silica Nanoparticles (ASNP) are among the most widely used nanomaterials in the food industry. They are not engineered to be on the nanoscale, but the traditional production processes result in the synthesis of primary nanoparticles which can variably agglomerate or aggregate depending on the conditions of production and use. ASNPs are usually considered safe for human health, and, recently, the former Panel on Additives and Nutrient Sources of the European Food Safety Authority concluded that “from the available database there was no indication for toxicity of silicon dioxide (E551) at the reported uses and use levels” [1]. However, the evaluation suffered from significant limitations (in the toxicological database, as well as in the physicochemical characterization of the materials employed in the in vivo studies [1]) and was carried out before the methodology for the nano-specific risk assessment became available [2]. Indeed, the assessment highlighted that the EU specifications for E551 are insufficient to adequately characterize silicon dioxide used as a food additive [1]. As far as hazard is concerned, ASNP toxicity was shown several years ago (see [3] for review) and was confirmed by a number of toxicological studies in vitro as well as in vivo (reviewed by [4]), including in recent long-term studies at realistic, low doses [5,6] and in investigations on the role of ASNP aggregation in toxicity [7]. An assessment of E551, specifically addressing the nanoscale nature of the material, highlighted that more insight into the health risk of AS in food was warranted [8].

ASNP interaction with cells of innate immunity is of particular interest. In terms of toxicity at the cellular level, Breznan et al. [9] have recently documented that ASNPs are cytotoxic and endowed with a pro-inflammatory activity in both murine and human macrophages. Moreover, they also showed that different preparations of ASNPs, of comparable sizes but different surface characteristics, have different toxicological properties, confirming that the identification of the physicochemical determinants of ASNP toxicity is not yet fully understood [4]. Winkler et al. demonstrated that food-grade, endotoxin-free ASNPs are able to activate the MyD88-pathway, leading to IL-1β induction in murine dendritic cells [10]. Macrophages are able to accumulate ASNPs upon prolonged or repeated exposure and, interestingly, upon co-exposure to bacteria or bacterial endotoxin, and ASNPs may lead to changes in macrophage activation via biological stimuli [11]. 

ASNP can be obtained following two methods of synthesis, the first requiring a high-temperature step to produce the pyrogenic form of NPs, while the other implies a low-temperature process to yield precipitated silica NPs. The ASNPs tested here are the NM-203 (pyrogenic) and NM-200 (precipitated), two preparations obtained by the JRC Nanomaterials Repository (Ispra, Varese, Italy). NM-203 and NM-200, which have been thoroughly characterized and are used as benchmark materials, are representative of the nanosized fraction of silicon dioxide contained in the E551 food additive. Distinct synthetic modalities are associated with subtle changes such as the external silanols’ density or spatial arrangement, which lead to major differences in the surface properties between pyrogenic and precipitated silica NPs [12]. Pyrogenic and precipitated ASNPs seem to exhibit different toxicities. In particular, pyrogenic ASNPs have been found to be toxic in several cell models [13] and in rat livers and kidneys [6,8,14], while the toxicity of precipitated ASNPs has been investigated in murine lungs, livers and kidneys [5,15,16], as well as in human THP-1 macrophages [17]. However, precipitated ASNPs only cause transient lung inflammatory changes following inhalation exposure [18] and are not endowed with a sizable hemolytic activity [19] or significant genotoxicity [20].

The risk of food grade ASNPs on human health through dietary intake was addressed by Dekkers et al. [21], considering two possible scenarios. The first one assumed that the material is taken up as dissolved silica, the second one predicted its gastrointestinal absorption as such (i.e., in particulate form). While no adverse effects were expected in the first scenario, there were too many uncertainties to allow a proper risk assessment in the case of the gastrointestinal absorption of NPs. A follow-up risk assessment, based on the evidence of ASNP biopersistence due to a negligible solubility and slow elimination from tissues [14,22], concluded that, notwithstanding the low gastrointestinal absorption, there was a potential for accumulation in the liver with a daily consumption at levels that were comparable to those detected in animal studies in which adverse effects were found [8]. 

Only few studies have evaluated ASNP accumulation following dietary exposure in vivo; moreover, in none of these was a direct toxicological comparison between pyrogenic and precipitated ASNPs performed [5,6,14,16], albeit toxicokinetic data produced within the Nanogenotox project highlighted that the two materials do not behave exactly the same [8]. When the two types of ASNPs were directly compared upon inhalation in rats, pyrogenic silicas were more inflammogenic than their precipitated counterparts [23], suggesting that they activated innate immunity cells more efficiently. This issue was directly investigated by Di Cristo et al. [24], who compared the effects on murine macrophages of NM-203 (pyrogenic) and NM-200 (precipitated), the two ASNP preparations used in this study. The results indicated that NM-203 adsorbed more proteins, elicited a larger oxidative stress and caused a higher pro-inflammatory activation than NM-200.

To ascertain if pyrogenic and precipitated ASNPs alter the inflammatory response to Pathogen Associated Molecular Patterns (PAMPs), we investigate here the effects of a pre-incubation with NM-200 or NM-203 on several parameters associated with the LPS-mediated activation of human THP-1-macrophages.

## 2. Materials and Methods 

### 2.1. Silica Nanoparticles 

Both precipitated (NM-200) and pyrogenic (NM-203) nanosilica materials (specific surface area 183.16 and 203.92 m^2^/g, respectively [25]), were provided by the JRC Nanomaterials Repository (Ispra, Varese, Italy). Before the experiments, ASNP powders were heated at 250 °C for 4 h in order to obtain LPS-free preparations. NPs were then dispersed in pure water supplemented with 0.05% Bovine Serum Albumin (BSA, Sigma-Aldrich, Milan, Italy) to obtain stock suspensions of 12.8 mg/mL, as described in Di Cristo et al. [24]. A thorough characterization of the materials has been provided previously [24,25]. As demonstrated in these earlier studies, the main physicochemical features of the two suspensions were comparable. In particular, the most relevant parameters were: primary particle size 14 ± 7 and 13 ± 6 nm; zeta potential −47.5 and −46.1 mV; and hydrodynamic radius in serum-supplemented culture medium 137.3 ± 11.5 and 138.3 ± 16.4 nm for NM-200 and NM-203, respectively [24,25]. 

### 2.2. Cell Culture and Experimental Treatments

The human acute monocytic leukemia THP-1 cell line was originally provided by the Cell Bank of the Istituto Zooprofilattico Sperimentale della Lombardia ed Emilia-Romagna (Brescia, Italy). The cells were cultured in high-glucose (4.5 g/L) DMEM (Dulbecco’s Modified Eagle’s Medium, Euroclone, Milan, Italy) supplemented with 2 mM glutamine (Sigma-Aldrich), antibiotics (100 U/mL penicillin and 100 μg/mL streptomycin) and 10% FBS (Fetal Bovine Serum, Gibco, Thermo Fisher, Milan, Italy). Before and during the treatments, the cultures were maintained in a humidified atmosphere of 5% CO_2_ in air at 37 °C and were passaged three times a week in 10-cm plates.

For the experiments, monocytes were seeded at a density of 3 × 10^5^ cells/cm^2^ into Falcon 24- or 96-well plates and differentiated into macrophage-like cells through a 48-h treatment with phorbol myristate acetate (PMA; 50 nM). The differentiated cells were washed three times with HBSS (Hank’s Balanced Salt Solution), maintained in culture for an additional 72 h in complete culture medium without PMA and then used for the experiments. For all the experiments, THP-1 cells were exposed for 24 h to NPs (range from 2.5 to 20 µg/cm^2^) and then challenged for further 24 h with LPS, added from a 10X stock solution in DMEM to reach the working concentration of 100 ng/mL.

### 2.3. Cell Viability and LDH Determination

The cell viability was assessed with the resazurin method on cells seeded on 96-well plates, as described in Bianchi et al. [26]. After the experimental treatments, the cell viability was tested by replacing the medium with a solution of resazurin (44 μM, Sigma-Aldrich) in serum-free DMEM. After 3 h, the fluorescence was measured (λ_EX_ = 515 nm; λ_EM_ = 586 nm) with a multimode plate reader Perkin Elmer Enspire (Waltham, MA, USA). Since nanomaterials could interfere with viability tests, a preliminary control was performed by incubating the dye with NPs only (20 μg/cm^2^) and then measuring the fluorescence. No fluorescence signal was detected above the background. The assessment of the LDH release in the culture medium was performed using the CytoTox 96^®^ Non-Radioactive Cytotoxicity assay kit (Promega Italia, Milan, Italy) following the manufacturer’s instructions.

### 2.4. Western Blot Analysis

The total cell lysates were obtained as described elsewhere [26]. The monolayers were rinsed with ice-cold PBS and then covered with 60 µL of Lysis buffer (20 mM Tris–HCl, pH 7.5, 150 mM NaCl, 1 mM EDTA, 1 mM EGTA, 1% Triton, 2.5 mM sodium pyrophosphate, 1 mM β-glycerophosphate, 1 mM Na_3_VO_4_, 1 mM NaF, 2 mM imidazole) supplemented with a protease inhibitor cocktail (Complete, Mini, EDTA-free, Roche, Monza, Italy). Samples were collected in Eppendorf tubes and then mixed with a proportional volume of sample buffer 4 × (250 mM Tris–HCl, pH 6.8, 8% SDS, 40% glycerol and 0.4 M DTT) before being boiled for 10 min. Samples were then loaded on 10% or 15% SDS-polyacrylamide gels, and proteins were separated for 1.5 h. After electrophoresis, the proteins were blotted on PVDF membranes (Immobilon-P, Millipore, Millipore Merck Corporation, MA, USA) for 1 h; the membranes were then incubated in TBS with a 10% blocking solution (Western Blocking Reagent, Roche) for 1 h and exposed overnight at 4 °C to primary antibodies diluted in TBS-T with 5% BSA. The used antibodies are listed in Table 1.

After three washes of 10 min each in TBS-T (50 mM Tris Base, 150 mM NaCl, pH 7.5), the membranes were exposed to the HRP-conjugated secondary anti-rabbit or anti-mouse IgG antibodies diluted at 1:10,000 in blocking solution for 1 h at room temperature (HRP, Cell Signaling Technology, Danvers, MA, USA). The visualization of protein bands was performed using the iBright™ FL1500 automated system (Life Technology, Thermo Fisher) after incubation with Immobilon Western Chemiluminescent HRP Substrate (Millipore, Merck). The band densities were analyzed with the iBrigth™ Analysis software 3.1.2 (Thermo Fisher).

### 2.5. RNA Extraction and Real Time PCR

Total RNA was isolated with the GeneJET RNA Purification Kit (Life Technology, Thermo Fisher) following the manufacturer’s instructions. 500 ng of total RNA of each sample were processed for reverse transcription. At the end of the process, aliquots of 25 ng/µL of cDNA were amplified in a total volume of 10 μL with the Power Up SYBR Green Master mix (Thermo Fisher), along with the forward and reverse primers (5 pmol each) reported in Table 2. Real-time PCR was performed in a Step One Plus apparatus (Thermo Fisher). 

For all the messengers to be quantified, each cycle consisted of a denaturation step at 95 °C for 15 s, followed by separate annealing (15 s) and extension (1 min) steps at a temperature characteristic for each pair of primers (Table 2). Fluorescence was monitored at the end of each extension step. A melting curve analysis was added at the end of each amplification cycle. The data analysis was conducted according to the relative standard curve method [27]. The expression data were reported as the ratio between each investigated mRNA and RPL15 mRNA.

### 2.6. Cytokine Determination

Both the IL-1β and TNF-α secretion in the culture media was determined with Quantikine^®^ ELISA kits (R&D System, Minneapolis, MN, USA). After 48 h of incubation under the conditions indicated for each experiment, 200 μL of culture medium were transferred to 96-well plates functionalized with anti-IL-1β antibody and incubated for 2 h at RT. The wells were then washed three times with 1X washing solution, and 200 μL of anti-Human IL-1β HRP-conjugated antibody were added to each well. After 1 h of incubation at RT, the samples were exposed for 20 min to 200 μL of substrate solution, before being incubated with 50 μL of stop solution and immediately read at 450 nm with a multimode plate reader Perkin Elmer Enspire.

### 2.7. Statistics 

The data were analyzed by Prism 5 (GraphPad, La Jolla, CA; USA). The values have been reported as the means ± SD of three independent experiments performed in multiple replicates. Differences between the groups were evaluated with a t-test or one-way ANOVA, as indicated in the figure legends. Differences were considered significant when *p* < 0.05.

## 3. Results

### 3.1. ASNPs Do Not Markedly Affect THP-1 Cell Viability 

**Figure 1:**
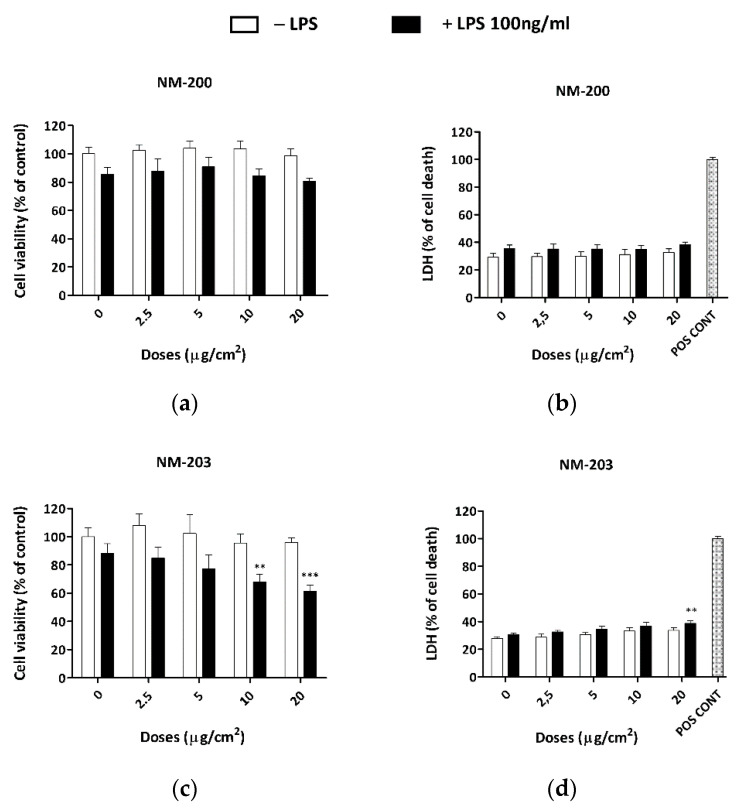
Effects of NM-200 and NM-203 on cell viability and cytotoxicity in THP-1 cultures. PMA-differentiated THP-1 cells were treated for 48 h with (**a**,**c**) NM-200 or (**b**,**d**) NM-203 (range 2.5–20 µg/cm^2^) in the absence (empty bars) or in the presence of LPS (100 ng/mL, solid bars) in the last 24 h of incubation. At the end of the experimental treatments, the cell viability was determined by (**a**,**b**) the resazurin method, while (**c**,**d**) the cytotoxicity was assessed from the LDH activity in the medium. Data are means of (**a**,**b**) five or (**c**,**d**) four independent determinations ± SD and are expressed as % of the control (no treatment) for (**a**,**b**), or as % of the positive control for cell death (**c**,**d**). The experiments were performed three times with similar results. ** *p* < 0.01; *** *p* < 0.001 vs. cells treated with LPS alone, one-way ANOVA. ‘Pos cont’, positive control.

To assess if ASNPs affect macrophage viability, we exposed differentiated THP-1 cells to increasing doses of either NM-200 (Figure 1a,c) or NM-203 (Figure 1b,d) for 48 h. In the last 24 h of treatment with ASNPs, the incubation was performed in the absence or in the presence of LPS (100 ng/mL). The results reported in Figure 1 show that, in the absence of LPS (empty bars), neither NM-200 nor NM-203 caused a significant decrease of cell viability or an increase in cell death at any tested dose. On the contrary, in the presence of LPS (solid bars), a moderate, dose-dependent decrease of cell viability, associated with an increase in cell death, was observed for the NM-203-treated cells but not for the NM-200-treated cells.

### 3.2. Autophagic Flux Is Blocked by Both Pyrogenic and Precipitated ASNPs

**Figure 2:**
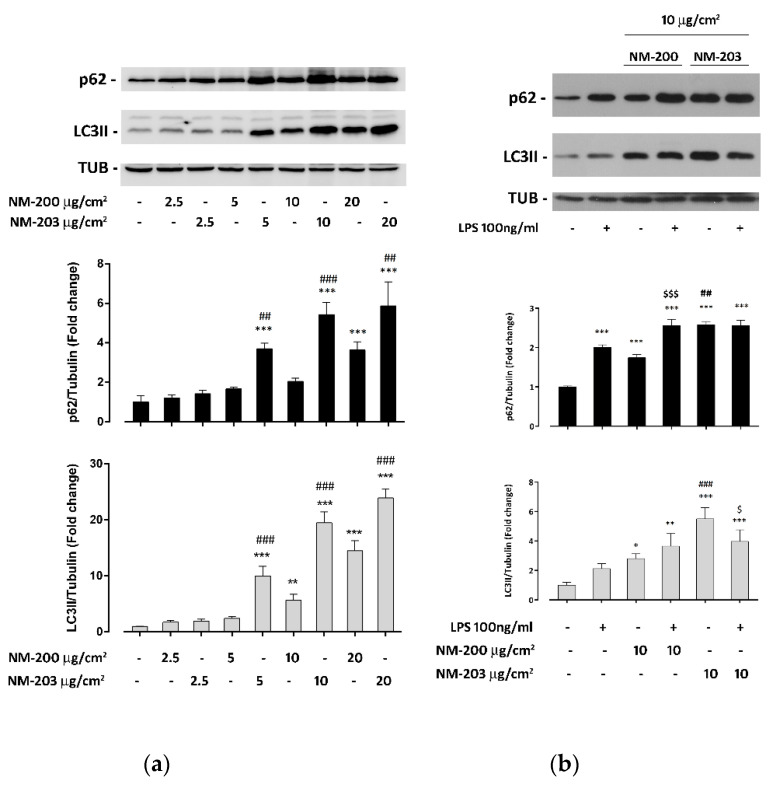
Impact of NM-200 and NM-203 on the autophagic flux. THP-1 cells were exposed for 48 h to (**a**) increasing doses of NM-200 or NM-203 or (**b**) to 10 µg/cm^2^ of NM-200 or NM-203 in the absence or in the presence of LPS (100 ng/mL) for the last 24 h. At the end of the experimental treatments, the cells were processed, and the protein expression of p62, LC3II and tubulin was determined. Bars report means of the relative abundance of LC3II (grey bars) or p62 (black bars) ± SD (n = 3). * *p* < 0.05; ** *p* < 0.01; *** *p* < 0.001 vs. untreated cells; $ *p* < 0.05; $$$ *p* < 0.001 vs. the same dose of NM-203 without LPS; ## *p* < 0.01; ### *p* < 0.001 vs. the corresponding dose of NM-200, one-way ANOVA.

It is known that ASNPs interfere with the autophagic flux in human endothelial cells as well as in murine macrophages [28,29]. To assess if NM-200 and NM-203 also had the same effects in human macrophages, we have evaluated the expression of LC3II and p62 protein in differentiated THP-1 cells treated with the two preparations of ASNP. As shown in Figure 2a, both ASNPs caused a dose-dependent increase of LC3II and p62 expression compared to the control. The lowest effective dose for a p62 and LC3II increase was 5 μg/cm^2^ with NM-203 and 10 or 20 μg/cm^2^, respectively, for LC3II and p62 with NM-200. NM-203 was significantly more effective than NM-200 at any tested dose. In cells pre-exposed to NM-200 for 24 h, the addition of LPS increased the expression of p62 but not that of LC3II (Figure 2b). With NM-203, instead, the addition of the endotoxin did not change p62 abundance, while it decreased that of LC3II (Figure 2b). LPS alone significantly augmented the p62 but not LC3II protein.

### 3.3. ASNPs Stimulate mTORC1 Activity and GS Expression

**Figure 3:**
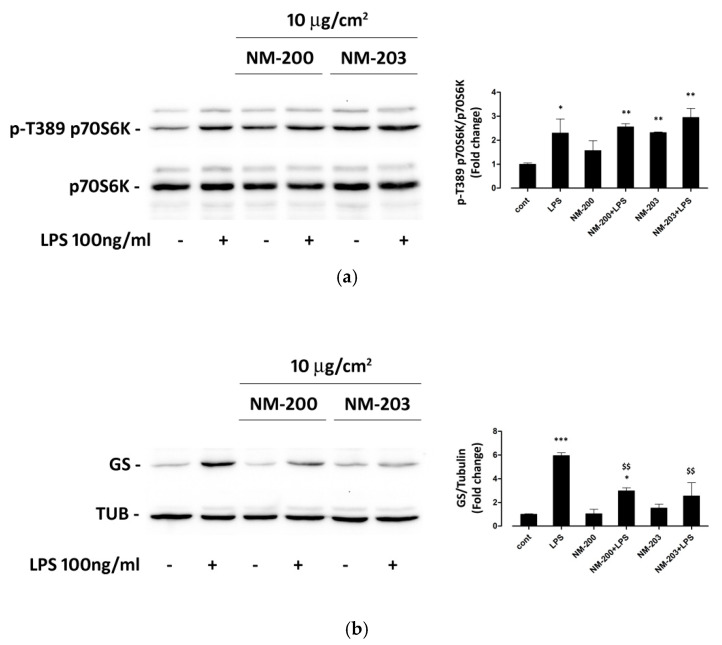
Effects of ASNPs on the mTORC1 activity and GS expression. THP-1 cells were treated as described above for Figure 2b. At the end of the experimental treatments, the expression of the indicated proteins was assessed. Bars report the means of the relative abundance of p-T389 pP70S6K vs. total pP70S6K (**a**) and GS vs. tubulin (**b**) ± SD (n = 3). * *p* < 0.05; ** *p* < 0.01; *** *p* < 0.001 vs untreated cells; $$ *p* < 0.01 vs LPS, one-way ANOVA.

The impact of pyrogenic or precipitated silica NPs on mTORC1 activity has never been investigated in macrophages. The results reported in Figure 3 reveal that the exposure to NM-203, but not to NM-200, significantly stimulated mTORC1 activity, as indicated by the increased abundance of p70S6K phosphorylated in T389, a mTORC1-specific site (Figure 3a). Furthermore, LPS stimulated mTORC1 activity, an effect that was neither inhibited nor increased by the pre-exposure to either ASNP. 

LPS raised the expression of Glutamine Synthetase (GS), the enzyme that produces Gln from Glu and ammonium (Figure 3b). In this case, the ASNP pretreatment significantly dampened the LPS-induced change, with a larger effect detected for NM-203.

### 3.4. ASNPs Do Not Affect the Expression of NFκB-Dependent Inflammatory Genes

**Figure 4:**
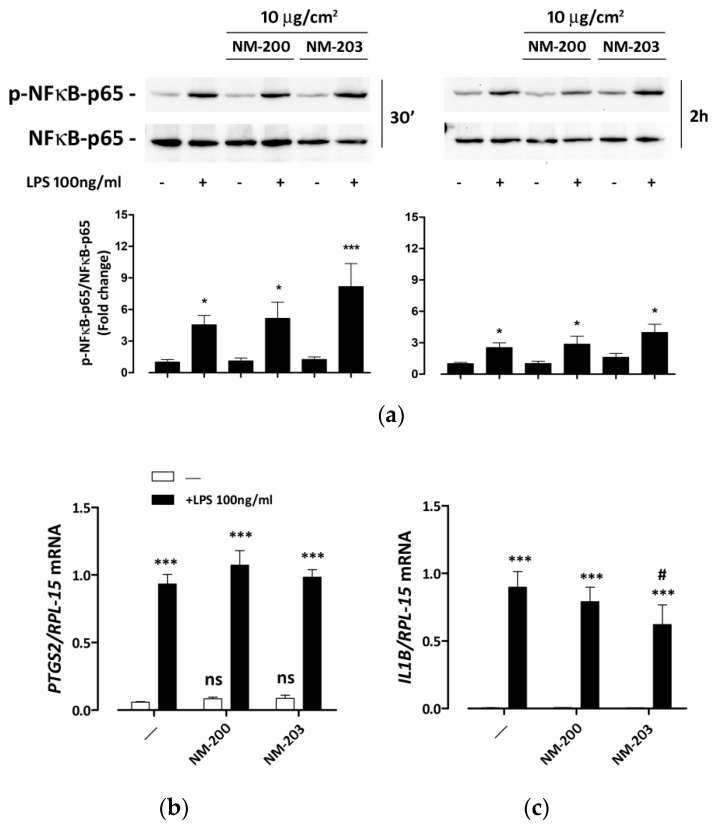
NFκB activation and induction of pro-inflammatory genes in ASNP-pretreated macrophages. THP-1 cells were incubated for 24 h with NM-200 or NM-203. The incubation was then prolonged with or without LPS. The expression of phosphorylated and total NFκB-p65, used as an activation marker and a loading control, respectively, was determined after (left, **a**) 30 min and (right, **a**) 2 h, while the expression of the mRNAs of (**b**) *PTGS2* or (**c**) *IL1B* was measured after 24 h. In (**a**), representative experiments are shown along with the means of the relative abundance of p-NFκB-p65 (bottom graph charts) protein ± SD (n = 3). * *p* < 0.05; *** *p* < 0.001 vs. untreated cells, one-way ANOVA. In panels **b** and **c**, data represent means of two independent determinations performed in duplicate ± SD. *** *p* < 0.001 vs. the same condition without LPS; # *p* < 0.05 vs. cells treated with LPS alone, two-tail t-test for unpaired data.

We then assessed if NM-200 and NM-203 affected the LPS-dependent activation of inflammatory response in THP-1 macrophages. After 30 min or 2 h of endotoxin exposure, ASNP-pretreated macrophages exhibited a LPS-dependent NFκB activation comparable to that detected in control cells (Figure 4a). Consistently, the LPS-dependent induction of *PTGS2* and *IL1B,* two inflammatory genes that are targets of NFκB, was not substantially modified by pre-incubation with ASNPs, although a small decrease of *IL1B* induction was observed only in cells pre-treated with NM-203 (Figure 4b).

### 3.5. ASNPs inhibit Caspase-1 Cleavage and Delay IL-1β Secretion

**Figure 5:**
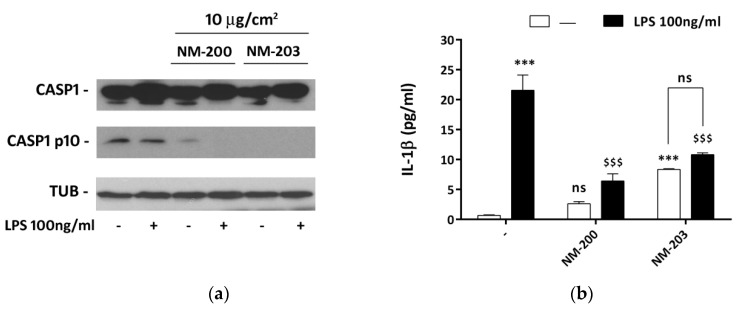
Caspase-1 cleavage and IL-1β secretion in ASNP-treated macrophages. THP-1 cells were incubated for 24 h with NM-200 or NM-203. The incubation was then prolonged with or without LPS. Cells were extracted for caspase-1 expression after 24 h of LPS treatment, while the culture media were collected for the determination of IL-1β after 2 h. In panel (**a**), a representative experiment is shown, performed twice with comparable results. In panel (**b**), data are shown as means of three independent determinations ± SD. *** *p* < 0.001 vs. control, untreated cells; $$$ *p* < 0.001 vs. LPS-treated cells not pre-exposed to ASNP; ns, not significant vs. the corresponding condition with LPS, one-way ANOVA.

The role of silica NPs in inflammasome activation was investigated by evaluating the expression of the cleaved form of caspase-1 and the secretion of IL-1β. A basal level of caspase-1 cleavage was already present in control, LPS- and ASNP-untreated THP-1 cells. After 48 h of exposure to ASNPs, the expression of the activated form of caspase-1 was markedly lowered by NM-200 and completely abolished by NM-203, although the expression of the uncleaved form was unchanged (Figure 5a). Suppression of activated caspase-1 was detected even in cells exposed to LPS in the last 24 h of incubation, although the expression of the uncleaved form apparently increased. 

LPS-untreated macrophages exhibited a very low, basal secretion of IL-1β. LPS caused a clear-cut stimulation of IL-1β already after 2 h of treatment (Figure 5b). In the absence of the endotoxin, exposure to NM-203, but not to NM-200, triggered a significant secretion of the cytokine. However, exposure to either NM-200 or NM-203 significantly dampened the LPS-dependent IL-1β secretion. Under these conditions, the stimulating effect of the endotoxin, calculated from the ratio between IL-1β secretion in the presence and in the absence of LPS, was 32 without pre-exposure to ASNP, dropping down to 2.5 for cells pre-exposed to NM-200 and to 1.3 for cells pre-exposed to NM-203, where the effect of LPS was no longer significant. It should be noted, however, that after 24 h of LPS incubation the levels of the cytokine secreted were comparable in cells pre-exposed to ASNPs and in control cells (data not shown).

## 4. Discussion

We recently showed that precipitated NM-200 and pyrogenic NM-203 ASNPs have different biological activities in murine macrophages [24]. In particular, NM-203 exhibited a higher capability to adsorb serum proteins and were more active than NM-200 in terms of cytotoxicity and inflammogenicity. In the present study, we characterized the effects mediated by the two ASNPs in human macrophages and assessed how the nanomaterials affected the cell responses to LPS, one of the most abundant environmental contaminants and the typical TLR4-dependent activator of innate immunity cells. The cell model we used consisted of human monocytic leukemia THP-1 cells, which acquire macrophage-like properties when differentiated by phorbol esters. Besides having a neoplastic origin, phorbol treatment may represent another limitation of this model, since the prolonged activation of protein kinase C could have several effects. For instance, it is known that phorbols promote caspase-1 activation [30], which was indeed already detectable in the absence of LPS in our conditions (Figure 5). However, the original procedure exploited in this study, with 3 d of wash out, should minimize other effects of the tumor promoter. Moreover, phorbol-differentiated THP-1 cells are widely used as a human macrophage model in toxicological studies on nanomaterials and, in particular, in studies concerning autophagy [31]. The assessment of the significance in vivo of the findings reported in this study will require a confirmation in a more physiological model, such as human primary macrophages.

A previous report demonstrated that both silica NPs slightly impair cell viability in several human and murine cell lines, with NM-203 being more active than NM-200 [32]. To assess the cytotoxic effects of ASNPs in THP-1 cells, we measured the cell viability and cytotoxicity after a 48-h exposure to a range of low doses of NM-200 and NM-203 (2.5–20 µg/cm^2^). The results reported in Figure 1 show that we observed a modest but significant, dose-dependent decrease of cell viability and an increase in cytotoxicity with NM-203, but not with NM-200, only when LPS was added during the last 24 h of incubation with ASNPs. 

In other cell models, ASNPs, as with many other engineered nanomaterials [33], are known to interfere with the triggering and progression of autophagy [4]. For instance, ASNPs activate autophagy in human endothelial cells [28,34,35], and this is considered a key step in the cell damage caused by the nanomaterial. Evidence in macrophages is scarce and somehow contradictory. Chou et al. [36] reported that mesoporous silica NPs trigger autophagy and, consistently, inhibit mTORC1 activity. Although autophagy activation by ASNPs was shown in murine macrophages [29,37,38], silica NPs produced a block, rather than an activation, of the autophagic flux in other cell models [28,39]. In most of those studies, ASNPs of different types were not compared. Such a comparison was actually performed by Breznan et al. [9], but with ASNPs endowed with characteristics that were clearly distinct from those of NM-200 and NM-203, such as a significantly lower negative zeta potential and, consequently, a stronger aggregation tendency. Most importantly, no specification was given by Breznan et al. on the production method that was adopted, which is exactly the issue addressed by our contribution. Moreover, while Breznan et al. described the inflammogenic potential of silica NPs per se, they did not address the very relevant question of how silica NPs can change the activation of human macrophages by naturally occurring compounds such as LPS. In the present work, we not only show that both ASNPs clearly produce a dose-dependent block of the autophagic flux, highlighted by a p62 accumulation, but also demonstrate that pyrogenic NM-203 are more effective then precipitated NM-200 in impairing the progression of autophagy. 

In several macrophage models [40,41,42], but not in THP-1 cells [43], LPS markedly triggered autophagy. Rather, under our conditions, LPS promoted the accumulation of p62 (Figure 2b). However, the effects of LPS and ASNPs on p62 levels were not additive, possibly indicating that the block of autophagy caused by the exposure to the nanomaterial occurs through the same mechanisms activated by LPS. Autophagy is one of the most important devices by which phagocytic cells eliminate pathogens present in the cytoplasm [44] and plays an essential role in the control of inflammatory response in the lung [45] and in the gut [46]. In particular, autophagy is required to avoid excessive inflammation on the basis of signals derived from gut microbiota [47]. Thus, the blockade of the autophagic flux mediated by ASNPs may have important functional consequences.

Since mTORC1 is one of the most potent negative regulators of autophagy, we assessed the activity of this kinase in THP-1 cells exposed to ASNPs. At a low, non-cytotoxic dose, NM-203 promoted p70S6K phosphorylation, indicating that pyrogenic ASNPs activated mTORC1, while the NM-200 effect was not significant (Figure 3a). This is, to our knowledge, the first report of mTORC1 stimulation after exposure to pyrogenic ASNPs of human macrophages. Silica NPs were found to also stimulate mTORC1 in other human cells, although no information about the type of ASNP eliciting such an effect was given in earlier studies [48]. Having noted this, it is indeed unclear why the marked kinase activation is apparently not associated with the suppression of autophagy triggering. It would be tentative to hypothesize that ASNPs mimic the overriding effect of intracellular *Salmonella* on the mTORC1-dependent inhibition of autophagy recently described by Liu et al. [41]. It should be stressed, however, that these experiments were performed at a single time point. Thus, we actually do not know if the autophagy block (Figure 2) occurs before, after or together with mTORC1 stimulation. As already described in THP-1 cells [49], LPS also stimulated mTOR activity (Figure 3a), but this effect was not additive with that of NM-203, nor was it changed by pre-exposure to NM-200, suggesting that the underlying mechanism might be the same.

On the contrary, ASNPs and LPS had opposite effects on the expression of Glutamine Synthetase (GS). LPS markedly induced GS protein expression, while pretreatment with NM-200 strongly attenuated the LPS effect, which was completely suppressed by NM-203 (Figure 3b). Both ASNP preparations did not affect the expression of GS in the absence of the endotoxin, suggesting a direct interference of the nanomaterial with the transduction of the LPS signal. In a recent study on monocyte-derived human macrophages, GS expression was linked to the activation status of the cells [50]. While a low GS expression would be associated with the M1 phenotype, its up-regulation has been considered a typical M2 feature [50]. Recent studies, performed on several models of innate immunity cells, supported this hypothesis, indicating that GS inhibition has a clear-cut pro-inflammatory effect [50,51,52,53]. If this were indeed the case, the increase in GS expression promoted by LPS would possibly constitute a kind of negative feedback to avoid an excessive stimulation of inflammatory cells. Thus, ASNPs, suppressing this LPS effect without preventing macrophage activation (Figure 4), would lessen this mechanism, possibly increasing LPS toxic effects. Interestingly, a significant cytotoxicity was only detected when LPS and NM-203 were together (i.e., when cells were stimulated but the GS increase was suppressed), even at the low doses of ASNP used. 

Neither ASNP influenced the phosphorylation levels of NFκB and the induction of its target genes by LPS (Figure 4). This negative result is biologically relevant, because it indicates that ASNPs do not generically interfere with all the pro-inflammatory activities of LPS-stimulated macrophages, and it allows one to attribute the effects of ASNPs on IL-1β secretion (Figure 5) to an interference with caspase-1 activation and not to a decreased transcription of the cytokine gene. Indeed, under our conditions, the expression of the cleaved form of caspase-1 was substantially suppressed by ASNPs (Figure 5), even in LPS-stimulated cells endowed with increased levels of the uncleaved form. Although recent data from Dalzon et al. showed that commercial ASNPs downregulated the caspase-1 protein expression in murine macrophages [54], no change in the uncleaved form of caspase-1 was found here after a 48-h exposure to ASNPs, suggesting instead that NM-203 and NM-200 interfered with the caspase-1 activation rather than its expression. The functional consequences of a caspase-1 defect are evidenced in Figure 5b, indicating that the short-term secretion of IL-1β was severely impaired in ASNP-treated cells, although the *IL1B* gene expression was comparable (Figure 4). However, after 24 h of LPS stimulation, the amount of secreted IL-1β was substantially comparable in ASNP-exposed or non-exposed cells, suggesting that caspase-1-independent mechanisms may have been at work. Although not investigated here, these alternative pathways may consist in the activation of non-canonical inflammosomes, which involve the pyroptosis-associated human caspases 4 and 5, already described in THP-1 cells [55]. This activation, along with the suppression of the LPS-dependent induction of GS expression, may account for the significant, although modest, toxicity exhibited by endotoxin-treated macrophages pre-exposed to ASNPs. 

As far as the identification of structural determinants underlying the different biological effects of precipitated and pyrogenic ASNPs is concerned, the increase of silanol groups at the surface of pyrogenic ASNPs was recently claimed as being an important determinant of toxicity [56]. However, in the same article, precipitated (“wet”) ASNPs had a high silanol density at the surface, despite being endowed with a low cytotoxicity [56]. Thus, it is unlikely that this parameter can explain why NM-200 and NM-203 have different efficacies in modulating autophagy, mTORC1 activity and GS expression. As demonstrated by Pavan et al. [57], the different interactions between various types of amorphous silica NPs and biological surfaces (in their case the red blood cell membrane) is mainly determined by the surface arrangement of silanols and siloxanes. Consistently, as demonstrated by Di Cristo et al. [24], even subtler surface variations, attributable to different production processes, may account for a different biological reactivity. For instance, NM-203 exhibit a higher capability to adsorb proteins from culture media then NM-200 do. Interestingly, high-density lipoprotein coronas are a major determinant of the differential cytotoxic action of silica NPs on monocytes and macrophages [58]. The relationship between the composition of the bio-corona and the biological effects of ASNPs should therefore be investigated in more depth in future studies in order to favor a regulatory distinction between pyrogenic and precipitated ASNPs.

To sum up, the results of the present study indicate that, in THP-1 macrophages: (a) ASNPs block autophagy, activate the mTORC1 complex and hinder caspase-1 activation; (b) ASNPs mimic some effects of LPS while impairing GS induction and IL-1β secretion promoted by the endotoxin; (c) pyrogenic ASNPs are distinctly more active than precipitated ASNPs, confirming that, for regulatory purposes, the two materials should not be considered the same. 

## 5. Conclusions

Although ASNPs are generally considered safe, we show here that low doses of these NPs modulate several important metabolic pathways in human macrophages and alter some of their responses to LPS stimulation. As far as autophagy, mTORC1 activity and GS expression are concerned, NM-203 cause larger effects than NM-200, confirming in a human macrophage model the greater bio-reactivity of pyrogenic ASNPs, previously documented by Di Cristo et al. in murine cells [24]. Moreover, when macrophages are pre-exposed to either ASNP, the short term LPS-dependent IL-1β secretion is markedly dampened, raising the possibility that the inflammatory response of these cells is delayed. The different biological reactivities of pyrogenic and precipitated ASNPs on innate immunity cells may be of particular relevance in the context of intestinal mucosa, where complex interactions among food components, mucosal tissue and microbiota occur [59]. The validation of this hypothesis will, however, require further investigations exploiting conditions and models closer to in vivo exposure conditions, as well as a more thorough characterization of ASNP hazard in relation to the physicochemical properties of the particles. A mechanistic understanding of the pathways leading to ASNP toxicity will definitely support an accurate risk assessment of dietary amorphous silica.

## Figures and Tables

**Table 1:** Antibodies used for the western blot.

Antibody	Host	Clonality	Dilution	Company
**anti-Caspase 1**	Rabbit	Monoclonal	1:500	Santa Cruz
**anti-Tubulin**	Mouse	Monoclonal	1:4000	Sigma
**anti-p-NFκB**	Rabbit	Polyclonal	1:1000	CellSignaling
**anti-NFκB**	Rabbit	Polyclonal	1:1000	CellSignaling
**anti-LC3 I/II**	Rabbit	Polyclonal	1:1000	CellSignaling
**anti-SQSTM1/p62**	Mouse	Monoclonal	1:10,000	Abcam
**anti-p-T389 p70S6K**	Rabbit	Polyclonal	1:1000	CellSignaling
**anti-p70S6K**	Rabbit	Polyclonal	1:1000	CellSignaling
**anti-GS**	Mouse	Monoclonal	1:1000	BD

**Table 2:** Primers used for the real-time PCR.

Gene	Forward	Reverse	T (°C)	Amplicon Size (bp)
**Interleukin-1 beta (*IL1B*)**	5’ ACA GAC CTT CCA GGA GAA TG 3’	5’ GCA GTT CAG TGA TCG TAC AG 3’	56 °C	127
**Cyclooxygenase-2 (*PTGS2*)**	5’ GGC TTC CAT TGA CCA GAG CAG 3’	5’ GCC GAG GCT TTT CTA CCA GA 3’	58 °C	194
**Ribosomal Protein L *(RPL15)***	5’ GCA GCC ATC AGG TAA GCC AAG 3’	5’ AGC GGA CCC TCA GAA GAA AGC 3’	56 °C	100
